# Editorial: Next generation sequencing and cancer: new steps towards personalized medicine

**DOI:** 10.3389/fgene.2025.1675753

**Published:** 2025-08-12

**Authors:** Erika Bandini, Clévia Rosset

**Affiliations:** ^1^ Biosciences Laboratory, IRCCS Istituto Romagnolo per lo Studio dei Tumori (IRST) “Dino Amadori”, Meldola, Italy; ^2^ Laboratory of Genomic Medicine, Experimental Research Service, Hospital de Clínicas de Porto Alegre (HCPA), Porto Alegre, Brazil; ^3^ Post-Graduation Program of Medical Sciences, Federal University of Rio Grande do Sul, Porto Alegre, Brazil

**Keywords:** Next generation sequencing (NGS), precision oncology, personalized medicine, cancer biomarkers, translational genomics

In the era of precision oncology, the scenario of cancer diagnostics, prognostics, and therapeutics has been revolutionized by the advent of Next generation sequencing (NGS). The ability to investigate cancer genomes and transcriptomes at remarkable depth has enabled the identification of novel mutations, fusion events, gene expression profiles, and non-coding RNA (ncRNA) signatures that contribute to tumorigenesis and therapeutic resistance. This Research Topic, next generation sequencing *and cancer: new steps towards personalized medicine*, has been conceived to explore how NGS technologies continue to drive forward the era of personalized oncology. This Research Topic, including original research and case reports, reflects the diversity and translational potential of NGS across tumor types and clinical contexts. The contributions cover key areas of precision oncology, ranging from biomarker discovery and drug sensitivity profiling to clinical case studies, that highlight the practical utility of NGS in guiding targeted therapies. Several studies emphasize the growing role of integrative bioinformatics and machine learning in finding out actionable insights from complex datasets. For example, Cui et al. applied topological data analysis and deep learning to uncover molecular drivers and predict therapeutic targets in melanoma, exploiting immune gene regulatory networks constructed from NGS data. Their computational approach exemplifies how artificial intelligence can bridge network-based analysis with precision oncology. Prognostic stratification is another critical application of NGS. Xu et al. performed a comprehensive analysis of programmed cell death (PCD) modalities in cholangiocarcinoma, integrating immune microenvironment features and drug sensitivity profiles. Their multi-dimensional approach stratified patients into prognostic subgroups and highlighted pathways that could inform personalized treatment strategies for this rare and aggressive cancer.

The role of ncRNAs, particularly circular RNAs (circRNAs) and microRNAs (miRNAs), continues to gain attention due to their regulatory impact in cancer. Zhang et al. investigated the role of circDOCK1 in colorectal cancer (CRC), combining microarrays and clinical data to characterize its expression and prognostic value. Similarly, Fuller et al. characterized miRNA signatures associated with gemcitabine resistance in pancreatic ductal adenocarcinoma (PDAC), a malignancy with poor prognosis and limited treatment options, shedding light on the molecular drivers of chemoresistance. Through miRNA sequencing of gemcitabine-sensitive and -resistant cell lines, the authors identified 97 differentially expressed miRNAs associated with critical cancer hallmarks such as proliferation, invasion, chemosensitization and apoptosis. Importantly, some of these miRNAs were further analyzed in patient samples, suggesting their potential as biomarkers for predicting recurrence or resistance, and highlighting how ncRNA profiling via NGS platforms can elucidate regulatory networks and identify novel therapeutic targets. Several case reports illustrate how NGS can guide personalized treatment in real-world settings. The case report by Dirven et al. provides a compelling example of a patient with stage IV-M1d melanoma harboring a class-2 *MAP2K1* mutation who experienced a durable intracranial and extracranial response to the MEK inhibitor trametinib combined with low-dose dabrafenib. NGS profiling enabled the identification of this actionable mutation, guiding targeted therapy that mitigated toxicity while achieving prolonged clinical benefit. This case highlights the utility of extending NGS beyond first-line decision-making, especially in patients with uncommon molecular subtypes. Similarly, Zhu et al. described a patient with Lynch syndrome who developed four distinct primary tumors over 26 years, a rare clinical course elucidated by targeted NGS, which revealed both a germline MSH2 mutation and distinct mutational profiles across tumors. Together, these individualized cases reinforce the central message of this Research Topic: personalized medicine is no longer a distant goal, but a current reality enabled by advanced sequencing technologies.

Collectively, the articles in this Research Topic exemplify how NGS is reshaping oncology across research and clinical practice, from molecular discovery to clinical decision-making. Several contributions explore the identification of novel fusion genes, regulatory RNAs, and resistance mechanisms, while others focus on integrating multi-omic data, refining prognostic models, or demonstrating NGS-guided therapeutic interventions. These studies highlight the critical need to integrate high-throughput sequencing data into clinical workflows, in order to optimize treatment strategies and improve patient outcomes. They also reveal the ongoing need for reliable bioinformatics pipelines, functional validation, and close interdisciplinary collaboration among researchers, oncologists, pathologists, and bioinformaticians. As NGS technologies continue to evolve, becoming faster, more affordable, and more accessible, they are increasingly positioned to serve as core components of routine oncology care. To fully realize their potential, this technological progress must be accompanied by collaborative efforts to standardize data interpretation, address ethical considerations around genomic information, and ensure equitable access to testing across all patient populations. This Research Topic reflects the rapid advancement in the integration of genomics into clinical practice, offering novel insights into the molecular drivers of cancer and illustrating how precision oncology is already transforming patient care.

